# *Culicoides* species community composition and feeding preferences in two aquatic ecosystems in northern Spain

**DOI:** 10.1186/s13071-022-05297-5

**Published:** 2022-06-11

**Authors:** Mikel A. González, Fátima Goiri, Sean W. J. Prosser, Aitor Cevidanes, Luis M. Hernández-Triana, Jesús F. Barandika, Paul D. N. Hebert, Ana L. García-Pérez

**Affiliations:** 1 Department of Animal Health, NEIKER-Basque Institute for Agricultural Research and Development, Basque Research and Technology Alliance (BRTA), Derio, Bizkaia Spain; 2 Centre for Biodiversity Genomics, University of Guelph, Guelph, ON Canada; 3 Vector-Borne Diseases Research Group, Virology Department-Animal and Plant Health Agency, Addlestone, UK; 4 Applied Zoology and Animal Conservation Research Group, Department of Biology, University of the Balearic Islands (UIB), Palma de Mallorca, Spain

**Keywords:** Barcoding, Biting midges, Host blood meals, Species richness, Freshwater habitats, Dynamic populations

## Abstract

**Background:**

Aquatic ecosystems provide breeding sites for blood-sucking insects such as *Culicoides* biting midges (Diptera: Ceratopogonidae), but factors affecting their distribution and host choice are poorly understood. A study was undertaken at two nature reserves in northern Spain to examine the abundance, species composition, population dynamics and feeding patterns of biting midges between 2018 and 2019.

**Methods:**

*Culicoides* were captured by light suction traps baited with CO_2_ and by sweep netting vegetation. Blood meals and species identification of blood-fed specimens were determined using cytochrome *c* oxidase I subunit (COI) DNA barcoding. Multivariate generalized linear models were used to evaluate the associations between the abundance of *Culicoides*, the species richness and other parameters.

**Results:**

The 4973 identified specimens comprised 28 species of *Culicoides*. These included two species reported for the first time in northern Spain, thus raising to 54 the number of *Culicoides* species described in the region. Specimens of all 28 species and 99.6% of the total specimens collected were caught in suction traps, while sweep netting vegetation revealed just 11 species and 0.4% of the total specimens. Midge abundance peaked in June/early July, with five species comprising > 80% of the captures: *Culicoides alazanicus* (24.9%), *Culicoides griseidorsum* (20.3%), *Culicoides poperinghensis* (16.2%), *Culicoides kibunensis* (10.7%) and *Culicoides clastrieri* (9.6%). DNA barcode analysis of blood meals from eight *Culicoides* species revealed that they fed on 17 vertebrate species (3 mammals and 14 birds). Species in the subgenus *Avaritia* were primarily ornithophilic, except for *C. griseidorsum* and *C. poperinghensis.* Host DNA from blood meals was successfully amplified from 75% of blood-fed females. A pictorial blood meal digestion scale is provided to accurately assess the blood-fed status of female *Culicoides.*

**Conclusions:**

The large number of different blood meal sources identified in the midges captured in this study signals the likely importance of wild birds and mammals (e.g. red deer and wild boar) as reservoir/amplifying hosts for pathogens. Available hosts are more exposed to being bitten by biting midge populations in aquatic ecosystems in late spring and early summer.

**Supplementary Information:**

The online version contains supplementary material available at 10.1186/s13071-022-05297-5.

## Background

Tiny blood-sucking midges (< 4 mm long) of the genus *Culicoides* (Diptera: Ceratopogonidae) are both biting pests and vectors of several viruses, filarial nematodes and protozoa of veterinary health relevance worldwide [1, 2]. At least 110 species of biting midges are included in the latest key for the Western Palearctic region [3], of which 84 are known from Spain [4–6]. In Europe, biting midges are not a threat to human health [1], but they do play an important role in transmission of both bluetongue virus (BTV) and Schmallenberg virus (SBV) to wild and domesticated animals [2, 7]. Ornithophilic *Culicoides* species also transmit avian parasites, such as the avian malaria parasite *Plasmodium* and the closely related genera *Haemoproteus* and *Leucocytozoon* [8, 9]*.*

A thorough understanding of host selection (i.e. feeding preferences) by *Culicoides* females is necessary to determine the complex relationship between hosts, vectors and pathogens [10]. The feeding preferences of midges in the subgenus *Avaritia* (*Culicoides obsoletus* group) and subgenus *Culicoides* (*Culicoides pulicaris* group) that frequently feed on livestock are much better studied than species with other feeding preferences [11–15]. In general, females of *Culicoides* exhibit a plastic feeding behavior determined by host preference, with most species feeding primarily on mammals or birds. Regardless of the preferred host, phylogenetically related species tend to feed on the same class(es) of vertebrates [16].

The role of *Culicoides* in the transmission of certain haemosporidian parasites remains poorly known as most surveillance and ecological studies have been conducted in agricultural settings with a focus on viral transmission to cattle or other livestock (goats, sheep and horses). As a result, the community of livestock-associated *Culicoides* species is relatively known in most of Europe [17, 18]. However, biting midges are also prominent in diverse semi-aquatic habitats, including swamps, marshes, forests, ponds, wet pastures, among others [19–22]. Wetlands and marshes are particularly valuable ecosystems for many animal and plant species, and serve as refuges and destination sites for native and migratory birds as well as for domestic and wild mammals. Consequently, there is high interest in preserving or expanding protected aquatic ecosystems, which also support many species of biting Diptera [23], including biting midges [24].

The propagation and transmission of pathogens via biting insects in wetlands and marshes have received little attention. Therefore, this study addresses this gap by examining *Culicoides* biting midges in these environments. We report data on the relative abundance and population dynamics of *Culicoides* and use DNA barcoding to identify the vertebrate species that served as the source of blood meals at various post-ingestion stages. The relevance of these findings is then discussed in relation to the monitoring and transmission of pathogens.

## Methods

### Study area

**Fig. 1:**
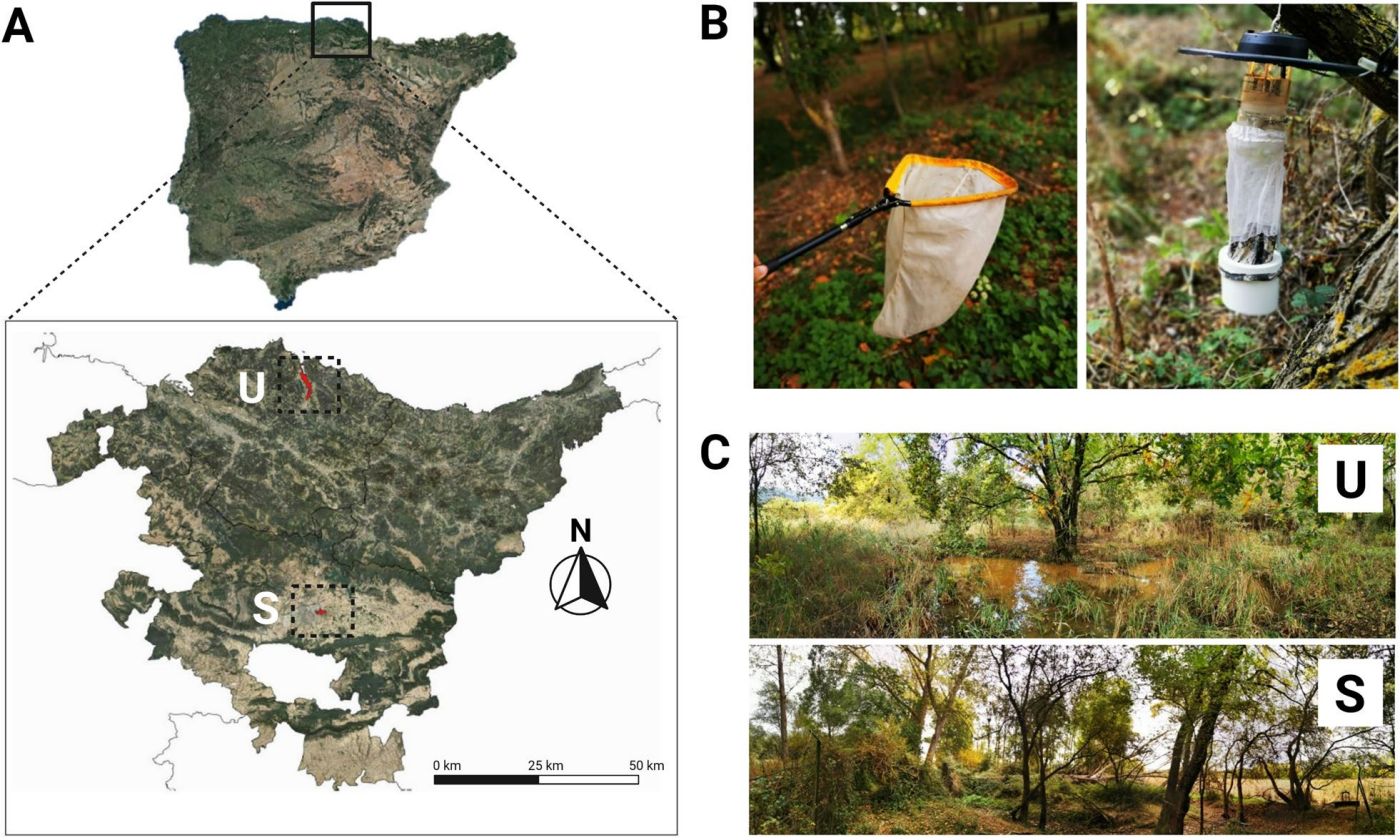
Location of the sampling sites and traps used to collect *Culicoides* biting midges. **a** Map of the Basque Country in northern Spain showing the two aquatic sites: Urdaibai (U) and Salburua (S). **b** methods of collection (sweep net and CDC traps, respectively).** c** Photograph of both settings

This survey was undertaken in the Basque region of northern Spain, at two nature reserves that are important to native and migratory birds (Fig. 1a). One of these areas is the Salburua wetland (Alava province; 42°51′ N, 02°39′ W) and the other is the Urdaibai marsh (Biscay province; 43°22′ N, 02°40′ E). Because these two sites are important wintering and migratory stopovers for multiple species of birds, they are registered in international programs, including the RAMSAR Convention (designating wetlands of international importance, especially as those of waterfowl habitats) and Natura 2000 (a network of nature protection areas within the European Union). These areas are also important public assets and include the Nature Interpretation Center (Salburua) and the Urdaibai Bird Center (Urdaibai), which together host 40,000–50,000 visitors annually. While the bird, mammal and herpetofauna at both sites have been well studied, prior work [25–27] on insects has only targeted the Coleoptera, Lepidoptera and Odonata orders; consequently, nothing is known about blood-sucking dipterans from these sites.

Salburua is a fenced park with a freshwater wetland located on the eastern outskirts of the city of Vitoria-Gasteiz. Spanning 2 km^2^, it is composed of several large pools, grasslands and oak groves bordered partly by an urbanized area with about 7000 residents. Approximately 290 vertebrate species (including 233 native and 4 exotic bird species) have been recorded at Salburua and a herd of about 120 non-native red deer was introduced for vegetation control (Luis Lobo, Centro de Estudios Ambientales, Vitoria-Gasteiz, personal communication). The climate is transitional between Oceanic and Mediterranean, rainfall is moderate (800 mm) and the average temperature is 11 °C, with more than 30 days of frost per year. The pools are fully recharged during the winter, then gradually become almost dry over the summer.

Urdaibai, a Biosphere Reserve designated by UNESCO in 1984, is located on the Bay of Biscay coast. It covers 220 km^2^ of small streams that merge into a large salt marsh surrounded by meadows, oak groves, woodlands and conifer plantations. Within the territory, there are 20 municipalities inhabited by about 45,000 people and some small cattle farms. Reflecting its large size and multi-ecosystem landscape, Urdaibai hosts at least 318 vertebrate species (approx. 250 birds), making it the most valuable ecosystem on the northern Spanish coast [28]. The reserve is constantly flooded, but the extent of flooding oscillates during the summer. The marsh ecosystem varies between freshwater and saline depending on location. The climate is Oceanic with abundant annual rainfall (1200 mm) and mild temperatures (average temperature of 14 °C) with less than 1 day of frost per year.

### Sampling sites

Five distinct environments were sampled from 1 July to 31 October 2018 (two habitats) and from 1 May to 31 October (three habitats) in each reserve. The five sampling sites in Salburua included: aquatic vegetation alongside a large temporary wetland pool (2018); a humid oak grove with streams (2018); grassland near a slow-flowing stream (2019); the margin of small shallow temporary ponds (2019); and a grassy poplar grove (2019). Sites in Urdaibai included: a humid woodland with intermittent ephemeral puddles (2018); vegetation alongside a permanent freshwater marsh (2018); the contact zone between a permanent freshwater habitat vegetated with bulrush; a mixed forest covered by ferns and brambles (2019); a temporary saline marsh with aquatic vegetation, primarily *Tamarix* spp*.* (2019); and mixed patches of trees and bushes admixed with streams, puddles and ditches near a livestock farm (2019).

### Carbon dioxide-baited CDC-traps

Centers for Disease Control and Prevention (CDC) 6 V battery-powered miniature traps (model 1212; John W Hock Co., Gainesville, FL, USA) equipped with incandescent light and baited with about 1.5 kg of dry ice that is released for 24 h through drilled polyethylene boxes, were employed to collect adult-biting midges as well as other blood-sucking Diptera (Fig. 1b). The traps were suspended at a height of 1–1.5 m and operated for 24 h periods (set up early in the morning and retrieved the next morning) every 2 weeks. Traps were hung on tree branches, where they were protected from sunlight and wind exposure. Collection pots were immediately transported to the laboratory and insects were stored at − 30 °C. A total of 55 carbon dioxide (CO_2_)-baited CDC traps were examined (16 in 2018 and 39 in 2019) in each wetland.

### Sweep net

Biting midges resting on vegetation were collected in the morning (between 9:30 and 11:00 a.m.) using a polyester mesh long-handled net (diameter: 38 cm, mesh size: 0.8 mm; BioQuip Products Inc., Rancho Dominguez, CA, USA) (Fig. 1b) by sweeping through grassy and shrubby vegetation (and on the foliage of bushes and shrubs) in a 25-m radius around each CDC trap. Sweeping was conducted very 2 weeks for approximately 4 min at each location. Sweep netting locations were limited by the type of habitat and time of year because flooding in the spring and densely vegetated areas in the summer restricted the effectiveness of this collection method. Collections were made by the same person each time, and collections were kept at – 30 °C for 20 min to kill the insects. A total of 55 sweep netting collections were examined (16 in 2018 and 39 in 2019) in each wetland.

### Morphological identification

Once in the laboratory, insects were sorted into major taxonomic groups. *Culicoides* midges were preserved in ethanol (70%), while other blood-feeding arthropods (data not shown) were preserved frozen for other studies. All biting midges were separated by sex and identified to species or species-group under a stereo microscope based on the wing pattern pigmentation, the shape and size of the third palpal segment (females) and other diagnostic morphological traits [29]. Males and females that presented atypical phenotypes, were damaged or were sibling species, or those whose identity was otherwise uncertain were mounted (after dissection of head, thorax + legs, wings and abdomen) on slides with permanent media (Hoyer´s medium), and species identification was achieved under a compound microscope. All species identifications were validated with the interactive identification key for Western Palaearctic *Culicoides* [3]. For species within the *Obsoletus* complex (*Culicoides obsoletus* and *Culicoides scoticus*), females were pooled due to the difficulties in discriminating these species morphologically, while males were identified based on diagnostic characters of the male genitalia [30, 31]. Blood-fed (*n* = 68) and gravid females (*n* = 12) were also mounted on slides, excluding the abdomen and thorax, which was used for molecular analysis.

### Molecular identification of blood-fed *Culicoides* species and their hosts

To maximize the sample size and assess the limit of detection of the molecular technique, all *Culicoides* females (*n* = 80) showing any trace of blood in their abdomen were analyzed. Specimens with successful amplification of host DNA (*n* = 53) were also DNA barcoded to confirm their species assignment. The specimens were also photographed to illustrate the five stages in blood meal digestion. To achieve these aims, the abdomen and thorax of each *Culicoides* midge were individually transferred to sterile vials (2 ml) and shipped on dry ice to the Centre for Biodiversity Genomics, University of Guelph (Guelph, Canada) for molecular analysis. Samples were processed following previously established methods [32, 33]. Briefly, DNA was extracted using a modified glass fiber technique [34]. The resulting DNA was used to ascertain the identities of the *Culicoides* species as well as of the vertebrate hosts upon which they had fed. *Culicoides* species were identified using standard DNA barcoding techniques, employing universal insect primers (C_LepFolF + C_LepFolR) [35] followed by Sanger sequencing. Traces were edited in CodonCode Aligner v9.0.1 and uploaded to the Barcode of Life Data System (BOLD). For vertebrate host identification, primers were designed to anneal to vertebrate but not insect DNA (C_BloodmealF1_t1 + Mod.Mamm.R_t1) [33] followed by next-generation sequencing on an Ion Torrent S5 Sequencer (Thermo Fisher Scientific, Waltham, MA). The resulting sequence reads were processed by first removing reads with a quality score (QV) < 20. Following primer/adapter trimming, reads in the expected size range of 125–250 bp were clustered into operational taxonomic units (OTUs) with a minimum identity of 98%. OTUs represented by at least 10 reads were compared to a reference library consisting of all vertebrate cytochrome* c* oxidase I gene (COI) barcode records on BOLD. Matches between an OTU sequence and a reference sequence were considered reliable only if at least 100 bp of the query sequence matched a reference with at least 95% homology. For each biting midge, all taxonomic matches were consolidated into a “unique taxonomic hit” table, with each hit supported by a total read count. Any taxonomic hits that occurred in negative control samples were proportionally subtracted from all other read counts, after which hits were only accepted as genuine if supported by at least 100 reads.

Full details for each *Culicoides* specimen, as well as their sequence information, can be found at the Barcode of Life Database (BOLD) within the “Human Pathogens and Zoonoses Initiative” Working Group 1.4. The Digital Object Identifier (DOI) for the publically available dataset on BOLD is 10.5883/DS-CULSPAIN. Accession numbers for all sequences were obtained from NCBI (OL702716-OL702759). Sequences for the *Culicoides* was analyzed in MEGA v.6 [36] and a neighbor-joining (NJ) analysis was performed using the Kimura 2-parameter distance. A Barcode Index Number (BIN) was assigned to all sequences longer than 500 bp, and each BIN was mapped according to species.

### Statistical analysis

Data on population dynamics were only plotted for 2019 because data were available for 6 months versus just 4 months for 2018. Generalized linear model (GLM) analysis was performed to evaluate the associations between *Culicoides* abundance (catch per trap per night) and species richness (S, number of species captured per trap per night) over the sampling period (July–October, shared trapping period for both years) with regards to aquatic ecosystems (Salburua and Urdaibai) and the year (2018 and 2019). Due to overdispersion of the data, a negative binomial GLM (NBGLM) was applied [37] using the MASS package [38]. For species richness, a GLM with Poisson error distribution and log-link function was used. The best model was selected with the “MuMIn” package of R software using the “dredge” function [39], which is based on the Akaike information criterion and corrected for sample size (AICc). The overall fit of the model was evaluated with a likelihood ratio test, comparing the best model with the null model. Statistical analyses were performed using R statistical software version 3.6.1 [40]. Relative abundance (RA) was calculated as the number of biting midges captured by one sampling method compared to the total number biting midges captured by both methods.

## Results

A total of 4973 *Culicoides* specimens were collected at the 10 sampling sites in 2018 and 2019 using suction traps baited with CO_2_ (Table 1) and sweep netting (Table 2). Other blood-sucking arthropods (excluding mosquitoes) were also identified, including eight specimens of *Chrysops viduatus* (Diptera: Tabanidae) and six specimens of *Simulium* spp. (Diptera: Simuliidae).

**Table 1:** *Culicoides* biting midges collected at two aquatic ecosystems in northern Spain with CO_2_-baited CDC-traps between 2018 and 2019

*Culicoides* species	CDC CO_2_-baited traps									
	Salburua				Urdaibai				Total	
	♂	♀	♂♀	%	♂	♀	♂♀	%	♂♀	%^a^
*C. alazanicus* Dzhafarov, 1961	22	952	974	25.8	11	251	262	22.3	1236	25.0
*C. griseidorsum* Kieffer, 1818	20	960	980	26.0	4	23	27	2.3	1007	20.4
*C. poperinghensis* Goetghebuer, 1953	24	766	790	20.9	2	13	15	1.3	805	16.3
*C. kibunensis* Tukunaga, 1937	9	390	399	10.6	4	127	131	11.2	530	11.7
*C. clastrieri* Callot, Kremer & Debuit, 1962	0	163	163	4.3	26	290	316	27.0	479	9.7
*C. festivipennis* Kieffer, 1914	46	254	300	8.0	2	34	36	3.1	336	6.8
*C. punctatus* (Meigen, 1804)	4	57	61	1.6	7	158	165	14.1	226	4.6
*C. newsteadi* Austen, 1921	1	0	1	< 0.1	5	54	59	5.0	60	1.2
*C. obsoletus* (Meigen, 1818)*/C. scoticus* Downes & Kettle, 1952^b^	0	20	20	0.5	6	34	40	3.4	60	1.2
*C. lupicaris* Downes & Kettle, 1952	0	9	9	0.2	1	28	29	2.5	38	0.8
*C. maritimus* Kieffer, 1924	0	0	0	0	5	31	36	3.1	36	0.7
*C. pictipennis* (Staeger, 1839)	13	20	33	0.9	2	0	2	0.2	35	0.7
*C. duddingstoni* Kettle & Lawson, 1951	0	11	11	0.3	0	5	5	0.4	16	0.3
*C. circumscriptus* Kieffer, 1918	0	2	2	< 0.1	0	8	8	0.7	10	0.2
*C. albicans* (Winnertz, 1852)^c^	0	0	0	0	0	9	9	0.8	9	0.2
*C. pallidicornis* Kieffer, 1919	0	0	0	0	0	9	9	0.8	9	0.2
*C. pulicaris* (Linnaeus, 1758)	0	2	2	< 0.1	1	6	7	0.6	9	0.2
*C. puncticollis* (Becker, 1903)^c^	0	8	8	0.2	0	0	0	0	8	0.2
*C. albihalteratus* Goetghebuer, 1935	2	1	3	< 0.1	1	3	4	0.3	7	0.1
*C. achrayi* Kettle & Lawson, 1955	0	2	2	0.1	1	3	4	0.3	6	0.1
*C. vexans* (Staeger, 1839)	0	6	6	0.2	0	0	0	0	6	0.1
*C. fascipennis* (Staeger, 1839)	0	4	4	0.1	0	0	0	0	4	< 0.1
*C. cataneii* Clastrier, 1957	0	0	0	0	0	2	2	0.2	2	< 0.1
*C. dewulfi* Goetghebuer, 1936	0	2	2	< 0.1	0	0	0	0	2	< 0.1
*C. gejgelensis* Dzhafarov, 1964	0	0	0	0	0	2	2	0.2	2	< 0.1
*C. parroti* Kieffer, 1922	0	2	2	< 0.1	0	0	0	0	2	< 0.1
*C. picturatus* Kremer & Debuit, 1961	0	0	0	0	1	1	2	0.2	2	< 0.1
Not identified	0	1	1	< 0.1	1	1	2	0.2	3	0.1
Total	140	3633	3773		80	1092	1172		4945	
S^d^			22				23		28	

All identifications were based on morphometric analysis

^a^The percentage of each species in the total catch

^b^Based on males, *C. obsoletus* accounted for five specimens (83.3%) and *C. scoticus* for one specimen (16.7%)

^c^First record for the Basque Country

^d^ Species richness

**Table 2:** *Culicoides* biting midges collected at two aquatic ecosystems in northern Spain with sweep nets between 2018 and 2019

*Culicoides* species	Sweep net									
	Salburua				Urdaibai				Total^a^	
	♂	♀	♂♀	%	♂	♀	♂♀	%	♂♀	%
*C. albihalteratus*	1	4	5	25.0	0	0	0	0	5	17.9
*C. griseidorsum*	1	4	5	25.0	0	0	0	0	5	17.9
*C. obsoletus/C. scoticus*	0	0	0	0	0	4	4	50.0	4	14.3
*C. festivipennis*	1	1	2	10.0	1	0	1	12.5	3	10.7
*C. poperinghensis*	0	3	3	15.0	0	0	0	0	3	10.7
*C. alazanicus*	0	2	2	10.0	0	0	0	0	2	7.1
*C. puncticollis*^b^	0	2	2	10.0	0	0	0	0	2	7.1
*C. kibunensis*	0	0	0	0	0	1	1	12.5	1	3.6
*C. maritimus*	0	0	0	0	1	0	1	12.5	1	3.6
*C. pallidicornis*	0	0	0	0.0	0	1	1	12.5	1	3.6
Not identified	0	1	1	5.0	0	0	0	0	1	3.6
Total	3	17	20		2	6	8		28	
S^c^			6				5		11	

All identifications were based on morphometric analysis

^a^The percentage of each species in the total catch

^b^First record for the Basque Country region

^c^Species richness

### *Culicoides* species community composition

**Fig. 2:**
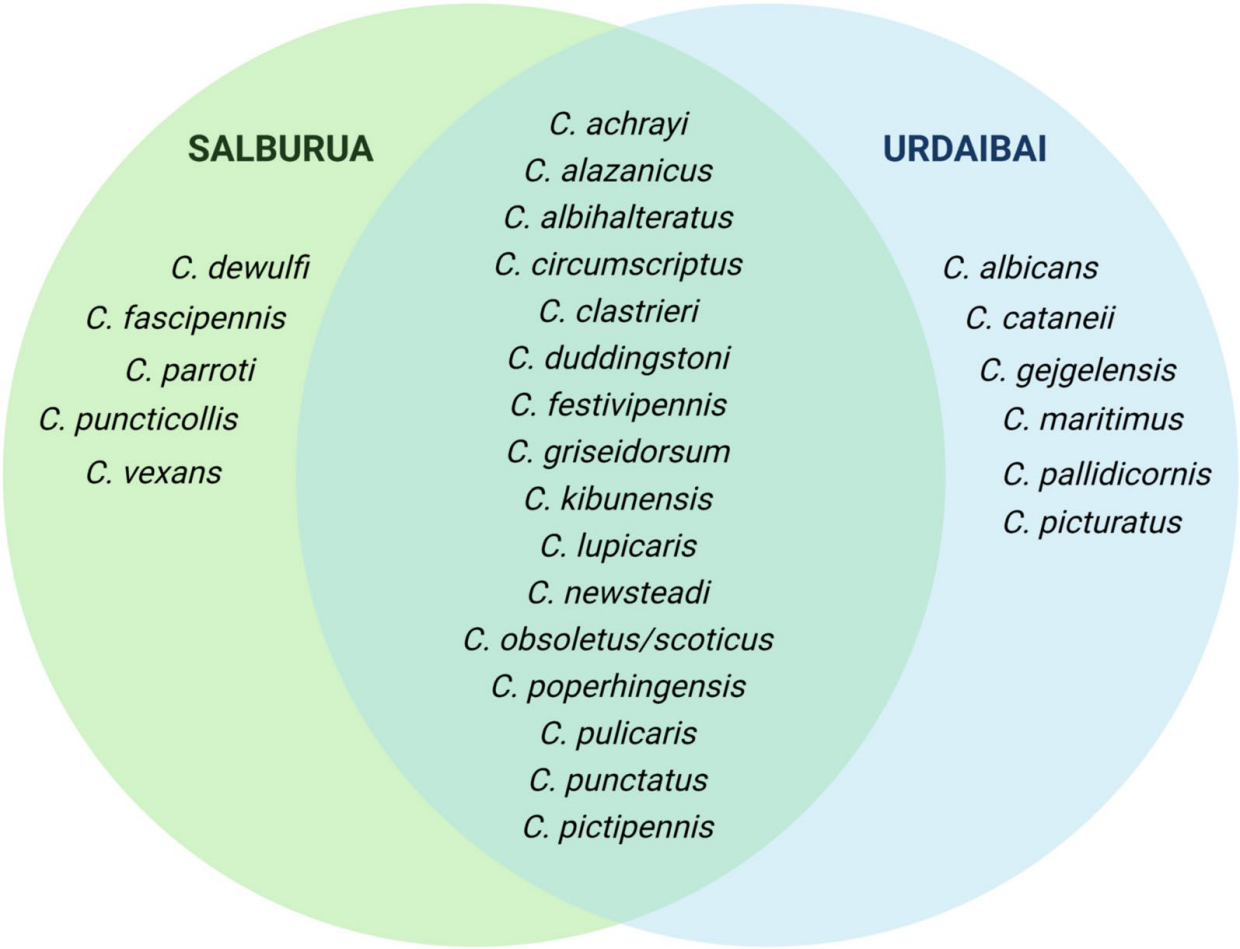
Venn diagram showing the presence of *Culicoides* biting midges in two aquatic ecosystems in northern Spain

Among the *Culicoides* specimens identified in this study, 4748 were female and 225 were male, and collectively they comprised 28 species (Tables 1, 2). Five species of the subgenus *Oecacta*, all known to feed on birds, dominated the assemblage. They included *Culicoides alazanicus* (*n* = 1238, 24.9%), *Culicoides griseidorsum* (*n* = 1,012, 20.3%), *Culicoides poperinghensis* (*n* = 808, 16.2%), *Culicoides kibunensis* (*n* = 531, 10.7%) and *Culicoides clastrieri* (*n* = 479, 9.6%). Species in the subgenus *Avaritia* (*C. obsoletus, C. scoticus* and *C. dewulfi*) were scarce (*n* = 66, 1.3%), while the remaining specimens represented 20 other species (Table 1). The three most common species at Salburua were *C. griseidorsum* (*n* = 985, 26.0%), *C. alazanicus* (*n* = 976, 25.7%) and *C. poperinghensis* (*n* = 793, 20.9%), whereas in Urdaibai they were *C. clastrieri* (*n* = 316, 26.8%), *C. alazanicus* (*n* = 262, 22.2%) and *C. punctatus* (*n* = 165, 14.0%) (Tables 1, 2). The distribution of *Culicoides* species varied between the two sites as they only shared 17 species (Fig. 2). Morphological traits were found to allow unequivocal species-level identifications for most slide-mounted specimens, excluding sibling species in the *Obsoletus* complex, which have an indistinct wing pattern. Two species (*Culicoides albicans* and *Culicoides puncticollis*) from Urdaibai and Salburua, respectively, represented first records from the Basque region.

### Population dynamics

**Fig. 3:**
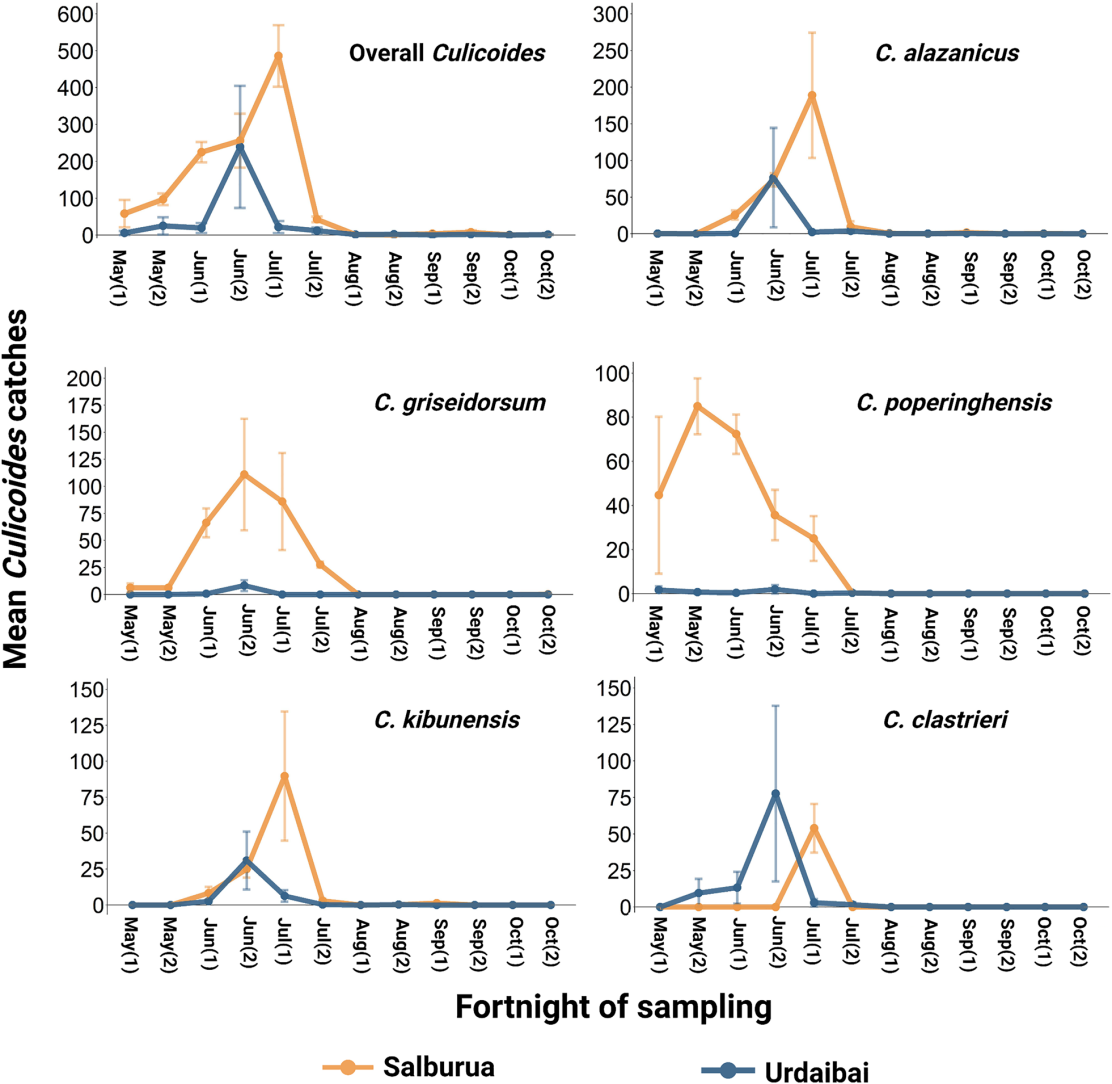
Seasonal dynamics of *Culicoides* species from 1 May to 31 October 2019 at two sites in northern Spain. Each point denotes the mean (± standard error) number of *Culicoides* caught per trap

Biting midges showed a single abundance peak in early summer (15 June–15 July). Midges from Salburua increased in number throughout the spring (May) until the maximum emergence in early July when 41% of all specimens were collected within an interval of 2 weeks. A similar trend was noted at Urdaibai, but peak abundance was 2 weeks later (i.e. capture of 76% of the total collection was concentrated in mid-late June) (Fig. 3). The most abundant species showed differing seasonal patterns. The earliest emerging species was *C. poperinghensis* (peaked in May) followed by *C. griseidorsum* (peaked in June) and *C. clastrieri*, *C. alazanicus* and *C. kibunensis* (peaked in early July) (Fig. 3).

### Analysis of variables affecting catch, abundance and species richness of *Culicoides*

Biting midges were mainly trapped by CO_2_-baited CDC traps (4945 specimens; relative abundance: 99.4%; species richness: 28) (Table 1) and rarely by sweeping (28 specimens; relative abundance: 0.6%; species richness: 11) (Table 2). Total *Culicoides* abundance varied between study sites, seasons and years. The abundance of biting midges was significantly higher in Salburua than in the marsh of Urdaibai (Table 3). The number of *Culicoides* specimens was also associated with the month (Table 3), with the highest catches in July, followed by a sharp decrease in August and a slight rebound in September. An annual variation was also observed, with a higher catch per trap in 2018 than in 2019. Species richness was higher at Salburua than Urdaibai, with more species during July followed by a steady decrease from August to October (Table 3).

**Table 3:** Summary of best models for total *Culicoides* abundance and species richness per trap and night

Variables	Abundance per trap/night			Species richness per trap/night		
	Estimate ± SE^a^	*Z*^*b*^	*P*-value	Estimate ± SE	*Z*	*P*-value
*Site*						
Urdaibai	Reference			Reference		
Salburua	1.22 ± 0.31	3.93	< 0.001	0.32 ± 0.14	2.23	0.025
*Month of sampling*						
July	Reference			Reference		
August	− 3.23 ± 0.41	− 7.78	< 0.001	− 0.90 ± 0.18	− 5.45	< 0.001
September	− 2.87 ± 0.40	− 7.06	< 0.001	− 1.12 ± 0.19	− 5.89	< 0.001
October	− 4.43 ± 0.47	− 9.44	< 0.001	− 2.06 ± 0.29	− 7.02	< 0.001
*Year*						
2018	Reference			Reference		
2019	− 0.68 ± 0.31	− 2.17	0.029	− 0.37 ± 0.14	− 2.65	0.008

^a^Standard error

^b^*Z*-statistic

### Molecular identification of *Culicoides* and their blood meals

Among the 53 *Culicoides* females that were barcoded to confirm their identity, 44 yielded a COI DNA barcode sequence. NJ analysis (Additional file 1: Phylogenetic analysis) showed that most barcode sequences clustered as expected based on morphological identifications. However, the COI barcode did not provide good resolution for *C. clastrieri *versus* Culicoides festivipennis* and *C. griseidorsum* versus* Culicoides pictipennis*, as these species grouped with the same BIN (BOLD: AEB9007 and ACV0334, respectively). Conversely, specimens of *C. kibunensis* were placed in two BINs that showed a mean divergence of 6.5%.

**Fig. 4:**
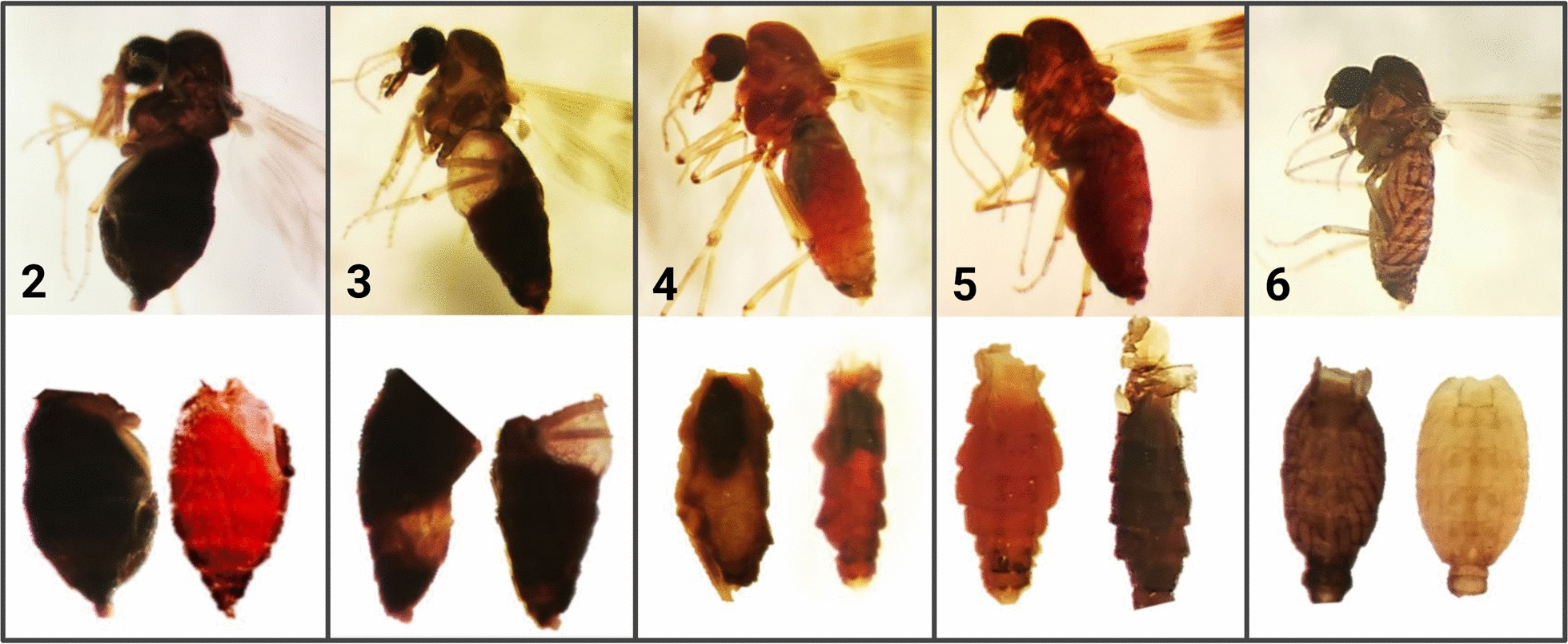
Different stages in the digestion of blood meals in *Culicoides* biting midges. The numbers 2–6 indicate the respective stages of blood meal digestion for *Culicoides* females. Stage 2: undigested (engorged abdomen showing intense dark-brown or fresh red tones) Stage 3: early digestion (engorged abdomen showing first signs of digestion with abundant dark blood). Stage 4: advanced digestion (abdomen showing advanced digestion but visible blood remains). Stage 5: pregravid (abdomen showing early egg formation mixed with a reddish tegument). Stage 6: gravid (engorged abdomen showing egg formation with no sign of blood). The upper row of images shows whole specimens of *Culicoides* and the lower row of images shows two different forms of the abdomens at the respective stage. Stage 1 (not shown) is the nulliparous stage

Among the trapped *Culicoide*s, only 1.6% (*n* = 68) were scored as blood-fed (containing at least visible remains of blood or red tegument, stages 2–5), while 12 more were scored as gravid. Figure 4 provides a pictorial blood meal digestion scale for *Culicoides* females. We identified the host DNA source for 75% (51/68) of the females in stages 2 to 5 and for 17% (2/12) of the gravid females. The success of blood meal identification was 100% for fully undigested blood meals (stage 2, *n* = 9) and early digested blood meals (stage 3, *n* = 10) but declined to 83% in females in stage 4 (20/24) and to 48% in those in stage 5 (12/25).

In total, 17 vertebrate species (3 mammals and 14 birds) were identified as the hosts for the eight *Culicoides* species with blood meals (Table 4). Of these specimens, 60% were found to have fed on avian hosts (32/53) and 40% on mammalian hosts (21/53). The most common mammal hosts were *Sus scrofa* and *Cervus elaphus*, while *Turdus* spp. and *Sylvia atricapilla* were the dominant bird hosts (Table 4). *Culicoides* midges showed a predominant affinity to feed on birds (subgenus *Oecacta*) with the exception of *C. griseidorsum* (Table 4).

**Table 4:** Origin of blood meals identified through DNA barcode analysis of eight species of *Culicoides* from two aquatic ecosystems in northern Spain between 2018 and 2019

*Culicoides*		Number	Avian hosts^a^	Mammalian hosts^a^
Subgenus	Species			
*Avaritia*	*C. obsoletus*	2	*Sylvia atricapilla* (1)	*Bos taurus (1)*
*Oecacta*	*C. griseidorsum*	15	–	*Cervus elaphus (10), Sus scrofa (5)*
	*C. alazanicus*	11	*Oriolus oriolus* (1),* Parus major* (1),* Pica pica* (1),* Cettia cetti* (1),* Carduelis carduelis* (1),* Turdus merula* (3),* Turdus philomelos* (1),* Sylvia atricapilla* (1),* Columba palumbus* (1)	─
	*C. festivipennis*	7	*Oriolus oriolus* (1),* Emberiza cirlus* (1),* Parus major* (1),* Pica pica* (1),* Sylvia atricapilla* (1),* Pyrrhula pyrrhula *(1),* Prunella modularis* (1)	─
	*C. kibunensis*	7	*Sylvia atricapilla* (3),* Turdus merula *(2),* Chloris chloris* (1),* Hippolais polyglotta* (1)	─
	*C. poperinghensis*	5	─	*Cervus elaphus (4), Sus scrofa (1)*
	*C. clastrieri*	5	*Sylvia atricapilla* (2),* Turdus merula* (1),* Columba palumbus* (1), *Parus major* (1)	─
	*C. duddingstoni*	1	*Carduelis carduelis* (1)	─
Total	8	53	14	3

^**a**^Number of hosts are given in parentheses

Blood-fed *Culicoides* midges were trapped primarily in June (*n* = 44) and July (*n* = 34) and only rarely in other months (May = 1; September = 1). The proportion of mammal versus avian hosts varied between the two sites, with a much higher proportion of mammalian hosts at Salburua.

## Discussion

An in-depth understanding of host-feeding preferences and the species composition of the *Culicoides* community at a site is a key component of disease surveillance programs and ecosystem health assessments. The investigation reported here revealed a high species richness and abundance of biting midges at two aquatic ecosystems in northern Spain. Interestingly, members of the *Obsoletus* complex, which are very abundant in most European agricultural settings [17], were very uncommon in these aquatic ecosystems, where ornithophilic *Culicoides* species were predominant, as has been reported in other studies [41]. As well, the most abundant species in this study (i.e. *C. alazanicus* and *C. griseidorsum*) showed a similar dominance in other aquatic ecosystems [42–44]. CDC-traps baited with dry ice captured more *Culicoides* than sweep netting, but the sampling effort (i.e. time) to collect midges by CDC-traps was much higher (24 h/trap vs. 4 min by sweep netting/sampling site). Although sweep netting is not often used to collect adult *Culicoides* [45], it can be effective for diurnal species, swarming aggregations and species that are rarely attracted to light, or to identify their potential resting places [46, 47].

Vector identification is key for the surveillance of arthropod-borne diseases as great differences in transmission capacity have been reported between closely related species [48]. Although a recently updated and illustrated interactive key has helped to identify *Culicoides* in Europe [3], the morphological identification of *Culicoides* species is difficult without training. DNA barcoding has been widely adopted as a tool for the rapid identification of insect species [49]. NJ analysis of the *Culicoides* barcode sequences generated in this study revealed that most conspecific specimens showed close sequence congruence, while members of different species showed deep divergence. However, specimens of *C. clastieri* grouped with specimens of *C. festivipennis* and were placed in the same BIN. Another study also reported low interspecific divergence between these two species [50], but they can be discriminated by morphometrics (number of spines in the *cibarium*, distribution of *sensillae coeloconia*, size of the R5 spot and color of thorax) [29]. *Culicoides griseidorsum* and *C. pictipennis* also shared barcode sequences but also could be distinguished by morphological characters. A converse situation was apparent for *C. kibunensis*, as specimens identified to this species belonged to two sequence clusters showing nearly 7% sequence divergence, suggesting the presence of sibling species.

In Europe, *Culicoides* midges in farmland settings are well-documented, and a marked variation in species abundances linked to trapping method, latitude and season has been shown [17]. However, much less data are available on *Culicoides* species in natural habitats. In contrast to farm-associated *Culicoides*, which show multiple abundance peaks from March to October [51], our study showed a single pronounced peak in early summer. This difference suggests that the most commonly trapped species (*C. alazanicus*, *C. griseidorsum*, *C. poperinghensis* and *C. kibunensis*) are univoltine, while species common in agricultural settings (*C. festivipennis, Culicoides newsteadi* and *C. obsoletus*) are multivoltine [17, 52]. This difference might be related to breeding sites, which are clearly different between agricultural and natural settings.

The factors responsible for the differences observed between 2018 and 2019 are uncertain, but they could reflect the shift in the sampling sites and/or more favorable climatic conditions in 2018. Threefold more midges were collected in the Salburua wetland, likely due to the abundance of freshwater habitats near the sampling sites [53–55], while the Urdaibai marsh was dominated by permanent bodies of saline water. The latter habitats may be less appropriate as developmental sites for the *Culicoides* species found in Europe, although salt marsh species are common in North America [56].

The use of blood meal DNA for host identification is constrained by its degradation and by the scarcity of blood-fed specimens. Prior work on mosquitoes has shown that the likelihood of recovering host DNA diminishes as digestion of the blood meal progresses until the formation of eggs [41–43]. However, this question is fairly unknown for *Culicoides*, and *Culicoides* females have typically been assigned to just two stages, i.e. fully/partly engorged or advanced digestion [11, 57]. To provide greater precision, we classified the digestion status of *Culicoides* into five categories reflecting the extent of digestion. Host identification was successful in all *Culicoides* females at stages 2–3, but it also worked, although at a lower efficiency, in specimens showing an advanced degree of blood digestion (stages 4–6) and in gravid *Culicoides* (stage 7). A South African study recovered host DNA from 19% of parous and 26% of gravid *Culicoides* specimens [58], supporting the value of analyzing all abdomens to assess host preferences. This is important because fully engorged females are rarely trapped by light-suction traps. By analyzing blood-fed females at all stages of digestion, we were able to identify the source of the blood meal in 75% of specimens, similar to success rates reported in other studies (44–91%) [15, 42, 59–62].

To understand and control the spread of pathogens through a community, the identity of both susceptible hosts and insects that vector the pathogens to them must be known. Our blood meal analysis of field-collected *Culicoides* females revealed differing host preferences, information essential for inferring their vector status [63]. The current study provides new perspectives because it is one of the few undertaken in natural settings as most earlier studies examined the *Culicoides* feeding preferences on a few domestic livestock host species. As might be expected, *Culicoides* in natural settings feed on a broad range of avian and mammal hosts. Our results support conclusions reached by Martínez de la Puente et al. [16] in that we found that members of the subgenus *Oecacta* (*C. alazanicus*, *C. festivipennis*, *C. kibunensis*,* C. clastrieri* and *Culicoides duddingstoni*) only fed on birds, with the exception of *C. griseidorsum* and *C. poperinghensis* which were primarily found to attack red deer (*C. elaphus*), in contrast to results reported in a previous study [43]. This is interesting because *Culicoides* species that feed on both wild and domestic ruminants act as bridge vectors [61]. Two specimens from the subgenus *Avaritia* (*C. obsoletus*) fed on a bird and a mammal. Those species that showed biting preferences for a wide range of birds deserve attention as some of them can transmit avian malaria [64].

One *Culicoides* specimen trapped at Urdaibai was found to have fed on cattle from a farm located 150 m from the trapping site and could only have reached the trap by crossing a dense forest patch. This relatively local dispersion after blood ingestion supports previous reports [11, 65]. Although some studies have reported longer dispersal distances [42], such cases likely reflect wind-mediated passive dispersal [66]. The distinctive differences in blood meals observed between the two study sites might reflect the differing availability of vertebrate hosts at each site. For example, the large population of red deer in Salburua likely provided easy access to hosts for *Culicoides*. No human-derived blood meals were recorded despite the presence of human settlements near both aquatic settings. This result was not a surprise as local *Culicoides* species are not attracted to humans, in contrast to the situation in other parts of Europe [60, 64, 67–70]. Most of the vertebrate hosts identified in this work are common in the study areas but some, such as the golden oriole (*Oriolus oriolus*), are summer trans-Saharan migrant species. Such instances exemplify how the use of blood meal analysis can be used to monitor rare species within a community [71, 72]. Finally, this survey contributes to knowledge on the species biodiversity of biting midges in the region as it raised the number of *Culicoides* species from 52 [29] to 54 species. Based on these results, the Basque Country region supports at least 64% of the total Spanish *Culicoides* fauna.

## Conclusions

This study expands our understanding of the *Culicoides* community at two sites in northern Spain, particularly with regards to host-use. Because this study is one of very few that has examined *Culicoides* in natural ecosystems as opposed to artificial agricultural settings, the information on host blood meals is more representative of the true host-range preference. The fact that most midges fed primarily on avian hosts has significant implications as it clearly suggests that *Culicoides* likely play an important role in vectoring avian parasites, particularly over early summer. In addition, some species fed on wild mammals (e.g. red deer and wild boar), which could act to amplify the host–vector cycle of several viruses, which in turn may affect livestock. Further studies should identify pathogens as well as the respective host(s) and vector(s) to help decipher their transmission dynamics.

## Supplementary Information

**Additional file 1:**Phylogenetic analysis (neighbor-joining method) of 44 *Culicoides* specimens based on the COI DNA barcode sequence.

## Data Availability

All data generated and analysed during this study are included in this published article.

## Abbreviations

AIC: Akaike information criterion
BIN: Barcode Index Number
BOLD: Barcode Of Life Database
CDC: Centers for Disease Control and Prevention
COI: Cytochrome *c* oxidase I gene
DOI: Digital object identifier
GLM: Generalized linear models
NBGLM: Negative binomial generalized linear model
NJ: Neighbor-joining tree
OTU: Operational Taxonomic Unit
QS: Quality score
RA: Relative abundance
BTV: Bluetongue virus
SBV: Schmallenberg virus
