# Supplementary figures and images for: Culicoides species community composition and feeding preferences in two aquatic ecosystems in northern Spain
Source: Parasit Vectors. 2022 Jun 11;15:199. doi: 10.1186/s13071-022-05297-5 (PMC9188056; doi:10.1186/s13071-022-05297-5)

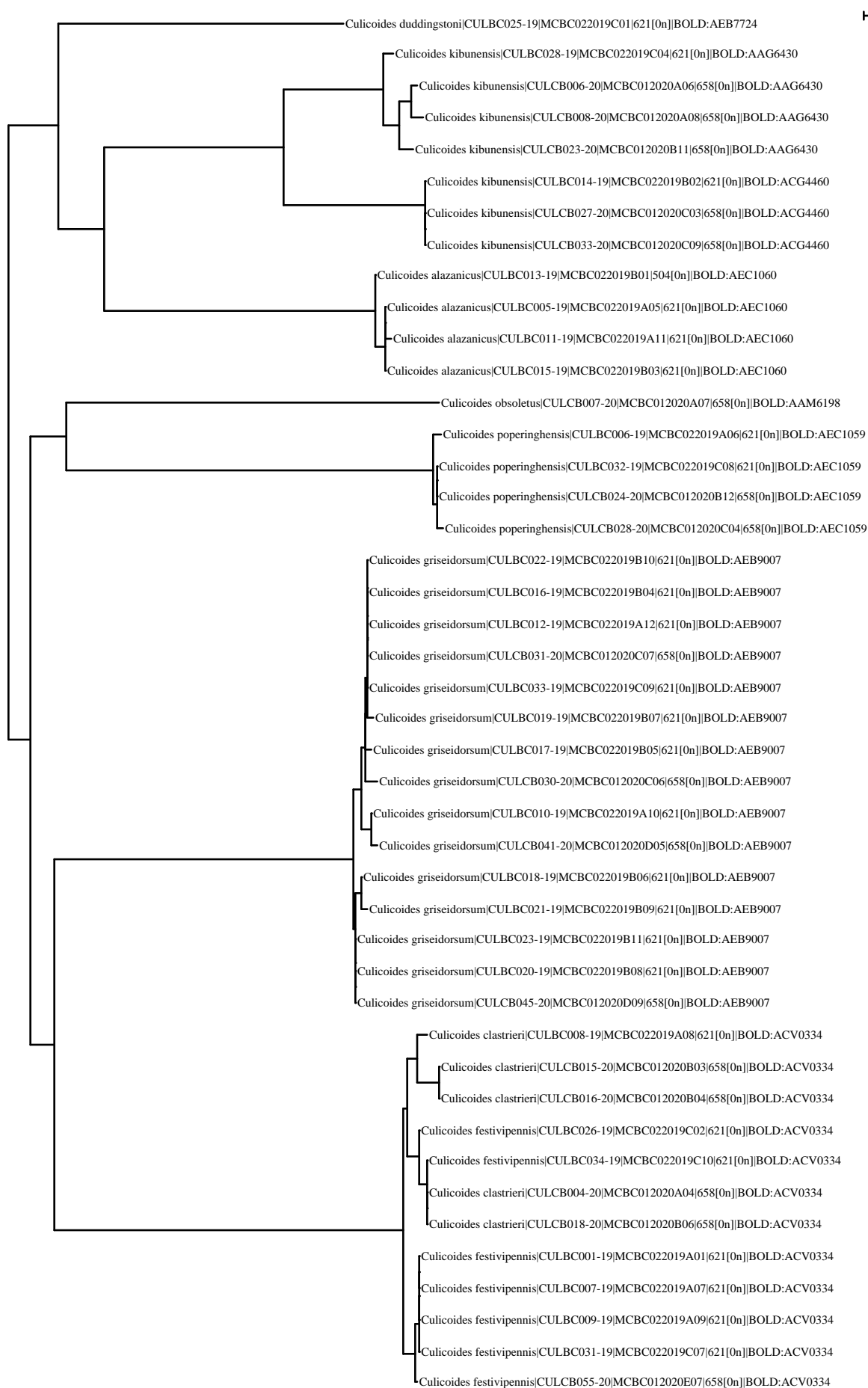

Supplement: Supplementary file 1 — Additional file 1:Phylogenetic analysis (neighbor-joining method) of 44 Culicoides specimens based on the COI DNA barcode sequence. [file 13071_2022_5297_MOESM1_ESM.pdf]
